# A systematic review of common genetic variation and biological pathways in autism spectrum disorder

**DOI:** 10.1186/s12868-021-00662-z

**Published:** 2021-10-09

**Authors:** Diego Alejandro Rodriguez-Gomez, Danna Paola Garcia-Guaqueta, Jesús David Charry-Sánchez, Elias Sarquis-Buitrago, Mariana Blanco, Alberto Velez-van-Meerbeke, Claudia Talero-Gutiérrez

**Affiliations:** 1 Neuroscience Research Group (NeURos), NeuroVitae Center for Neuroscience, School of Medicine and Health Sciences, Universidad del Rosario, Carrera 24 No. 63C-69, 111221 Bogotá D.C., Colombia; 2 NeuroVitae Center for Neuroscience, School of Medicine and Health Sciences, Universidad del Rosario, Carrera 24 No. 63C-69, 111221 Bogotá D.C., Colombia

**Keywords:** Autism Spectrum Disorder, Pathophysiology, Polymorphisms, Genetics, Synapsis

## Abstract

**Background:**

Autism spectrum disorder (ASD) is a complex neurodevelopmental condition characterized by persistent deficits in social communication and interaction. Common genetic variation appears to play a key role in the development of this condition. In this systematic review, we describe the relationship between genetic variations and autism. We created a gene dataset of the genes involved in the pathogenesis of autism and performed an over-representation analysis to evaluate the biological functions and molecular pathways that may explain the associations between these variants and the development of ASD.

**Results:**

177 studies and a gene set composed of 139 were included in this qualitative systematic review. Enriched pathways in the over-representation analysis using the KEGG pathway database were mostly associated with neurotransmitter receptors and their subunits. Major over-represented biological processes were social behavior, vocalization behavior, learning and memory. The enriched cellular component of the proteins encoded by the genes identified in this systematic review were the postsynaptic membrane and the cell junction.

**Conclusions:**

Among the biological processes that were examined, genes involved in synaptic integrity, neurotransmitter metabolism, and cell adhesion molecules were significantly involved in the development of autism.

**Supplementary Information:**

The online version contains supplementary material available at 10.1186/s12868-021-00662-z.

## Background

Autism spectrum disorder (ASD) is a neurodevelopmental condition characterized by persistent deficits in social communication and interaction, in addition to various restrictive and repetitive behaviors [1–3]. Several twin and family-based studies have shown that ASD is highly heritable. However, the specific genetic mechanisms that underlie this heritability are not yet fully understood [2–6]. Increasing evidence suggests that the bulk of genetic risk associated with ASD may be due to single nucleotide polymorphisms (SNPs) and copy number variants (CNVs), rather than specific single-gene mutations or syndromic conditions, which constitute only approximately 5–15% of ASD cases [5].

The identification of common genetic variants associated with the etiology of ASD has increasingly been used as a strategy to understand the mechanisms underlying this condition. Multiple family-based case-series and case–control studies around the world have identified common genetic variants that account for a portion of our understanding of ASD. However, these studies are typically directed towards the identification of common genetic variants in one or a few specific genes associated with ASD. The larger picture of the pathophysiology of ASD requires this information to be integrated into one or multiple genetic and molecular pathways that can provide further insights into the mechanisms that drive ASD.

The proper functioning of the brain and central nervous system requires appropriate synaptic morphology and function [7, 8]. Common genetic variations in candidate genes and their interactions within multiple biochemical and cellular pathways have revealed that synaptic and neurotransmitter dysfunction are essential components in the development of ASD [7–9]. Indeed, various neurologic and psychiatric diseases, including schizophrenia, Alzheimer’s disease, and ASD, have been associated with synaptic and neurotransmitter dysfunction [8].

Several studies have addressed the role played by synaptic alterations in the etiology of ASD [8–11]. A significant proportion of the genetic variation that has been described in ASD patients has been associated with genes that are either directly or indirectly involved in synaptic structure and function [9, 10]. This review aims to provide an overview of the known genetic variations and their contributions to biological pathways involved in ASD such as synaptic and neurotransmitter dysfunction.

## Methods

### Study design

This study was performed as a systematic review of the literature. The protocol registration can be found under the PROSPERO ID CRD42020206689.

### Search strategy

To identify relevant articles for inclusion in this systematic review, a search was performed in PubMed using the following MeSH terms: (“Autism Spectrum Disorder,” OR “Autistic disorder,” OR “Rett Syndrome”) AND “Polymorphisms (Genetics).” Additionally, the search results were filtered using three criteria: (1) studies published during the last 20 years (2000–2020), (2) studies performed only in humans, and (3) studies written in English. Additionally, the references for each included paper were reviewed to identify relevant citations that were retrieved manually using Google Scholar and PubMed.

### Study selection

Articles were included in our systematic review if they fulfilled the following study criteria: (1) Patients with an ASD diagnosis, according to the Diagnostic and Statistical Manual of Mental Disorders criteria (DSM-IV or DSM-5), the International Classification of Diseases (ICD) criteria, Childhood Autism Rating Scale (CARS), Autism Diagnostic Interview-Revised (ADI-R), or other scales specifically designed to assess ASD. (2) Genetic testing strategies to identify SNPs, CNVs, or other genetic variants in either one or multiple ASD candidate genes. (3) Case–control studies, family-based case series, or case reports performed using human subjects. Patients with tuberous sclerosis or fragile X syndrome were excluded since these conditions are associated with specific mechanisms and genetic variants.

During the study selection process, articles were filtered first by title and then by reading the abstracts. This process was performed by the leading author, and the results were discussed with all the members of the team during weekly meetings. Disagreements were resolved by discussion or independent review by a second author.

### Quality assessment

Quality assessment was performed using the Joanna Briggs Institute clinical appraisal tools. Studies were divided among all the team members, and the results of the quality assessment were discussed during weekly meetings. The reasons for article exclusion based on quality were always explicitly considered. In case of doubt, the article was independently reviewed by a second author. Any additional disagreement was resolved by discussion among the members of the team.

### Data extraction

Data were extracted from the included articles to obtain the following information: study design, characteristics of the studied population, study objective, genetic tests performed, genes studied, and variations identified. A data set of genes associated with ASD and common genetic variations was generated for the over-representation analysis. Genes and genetic variants were included in the data set when a significant positive association was identified between the studied SNP or CNV and the presence of ASD.

### Over-representation analysis

The biological interpretation of identified genes was performed using the Functional Annotation Tool from the Database for Annotation, Visualization, and Integrated Discovery (DAVID) [12, 13]. Within DAVID, the Kyoto Encyclopedia of Genes and Genomes (KEGG), Gene Ontology (GO) Biological Process (BP), and GO Cellular Component (CC) databases were chosen for the over-representation analysis. The data set of genes obtained was analyzed against the human genome background to identify whether the proportion of genes within the data set was higher than the one expected by chance in each of the biological pathways of the selected databases. For this analysis, the EASE score was set to be lower than 0.05 in order to define statistically significant enrichment. This value was calculated as a conservative modified Fisher’s exact p-value that removes one gene within each given category and then calculates the exact Fisher probability for that category. As a result, the EASE score penalizes the significance of categories with few genes, which favors more robust categories in the over-representation analysis [14]. Bonferroni correction using the Bonferroni Šidák p-value provided by DAVID was also included. An additional over-representation analysis of biological pathways was conducted using the Reactome database, [15] and Fisher’s exact p-values were calculated to complement the results obtained in DAVID.

## Results

### Search results and study characteristics

**Fig. 1:**
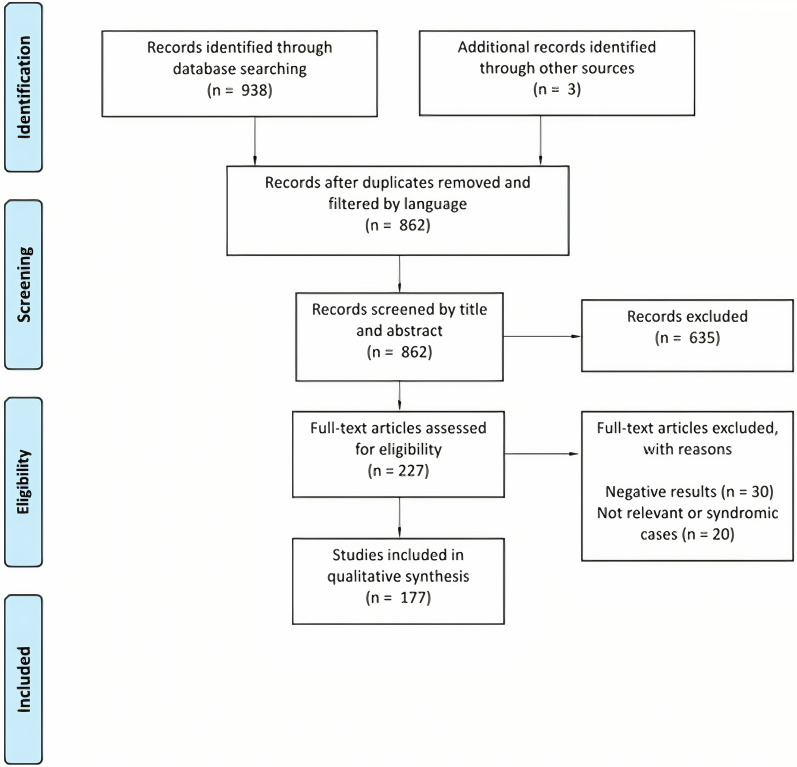
PRISMA flow diagram for the studies included in the systematic review

A total of 941 articles were identified using the initial search criteria, including those manually retrieved. After removing duplicated papers and articles written in other languages, 862 articles remained. These records were screened by title and abstract, and 227 articles were selected for full-text assessment. From these articles, 30 were excluded due to negative results, and 20 were excluded due to the inclusion of syndromic or irrelevant cases. Finally, 177 studies were included in this qualitative systematic review (Fig. 1). During data extraction, a gene set composed of 139 genes was generated, each associated with several SNPs. Other genetic variants identified within the selected studies included CNVs and microdeletions. Further information regarding each study and the genes identified for inclusion in the gene set can be found in the Additional file 1 for this paper.

Almost all of the included studies were either case–control studies or family-based case series. These studies were conducted among different populations; the Chinese Han ethnicity was the most commonly studied population, appearing in 35 (19.77%) articles. Other populations that were frequently studied included the Autism Genetic Resource Exchange from the United States (30 articles; 16.94%), the Iranian population (10 articles; 5.64%) and the Korean population (9 articles; 5.08%). The studies also examined cohorts from Egypt, Iran, Saudi Arabia, and several European countries. In most cases, the diagnosis of ASD was based on the DSM-IV or DSM-5 criteria; however, several other tools, such as the ADI-R and CARS, were also used.

### Results of the over-representation analysis

An over-representation analysis was performed using DAVID and the gene set that was generated from the data extraction process. The most significant terms that were enriched in our gene set using the KEGG pathway database are shown in Table 1. Most of the enriched pathways were associated with either a type of chemical synapse or a process associated with synaptic function. Some of the genes associated with these pathways included genes that encode neurotransmitter receptors and their subunits, such as serotonin receptors (*HTR2A* and *HTR3A*), γ-aminobutyric acid (GABA)-A receptors (*GABRA4*, *GABRA5*, *GABRB1*, *GABRB3, GABRG2*, and *GABRG3*), glutamate receptors (*GRIN2A*, *GRIN2B*, *GRIK2*, *GRIK5* and *GRM7*), dopamine receptors (*DRD1*, DRD2 and *DRD3*), and oxytocin receptors (*OXTR*). Neurotransmitter metabolism genes were also identified, including dihydroxyphenylalanine (DOPA) decarboxylase (*DDC*) and monoamine oxidase genes (*MAOA* and *MAOB*).

**Table 1:** Results of the over-representation analysis using the KEGG Pathway database

Term	#	Genes	p-value	Bonferroni
Serotonergic synapse	13	*HTR2A, HTR3A, CACNA1C*, *DDC*, *GABRB1, GABRB3, MAOA, MAOB, PLA2G4C*, *PTGS2*, *PRKCB*, SLC6A4, TPH2	3.4 × 10^−8^	3.2 × 10^−6^
Neuroactive ligand-receptor interaction	19	*HTR2A, ADRB2*, *AVPR1A*, *DRD1, DRD2, DRD3, GABRA4, GABRA5, GABRB1, GABRB3, GABRG2, GABRG3, GRIN2A, GRIN2B, GRIK2, GRIK5, GRM7, OXTR, VIPR2*	3.6 × 10^−8^	3.2 × 10^−6^
Calcium signaling pathway	14	*HTR2A, ATP2B2*, *ADRB2, AVPR1A, CACNA1C, CACNA1G*, CHRNA7, *DRD1, GRIN2A, NOS2, OXTR, PRKCB, SLC25A6*	9.9 × 10^−7^	3.0 × 10^−5^
Dopaminergic synapse	12	*CACNA1C, DDC, DRD1, DRD2, DRD3, GRIN2A, GRIN2B, MAOA, MAOB, PRKCB, PPP2R5D*, *SLC18A1*	1.4 × 10^−6^	3.5 × 10^−5^
GABAergic synapse	9	*ABAT, CACNA1C, GABRA4, GABRA5, GABRB1, GABRB3, GABRG2, GABRG3, PRKCB*	2.1 × 10^−5^	4.8 × 10^−4^
Glutamatergic synapse	10	*SHANK2, SHANK3, CACNA1C, GRIN2A, GRIN2B, GRIK5, GRM7, HOMER1*, *PLA2G4C, PRKCB*	2.7 × 10^−5^	5.3 × 10^−4^
Retrograde endocannabinoid signaling	9	*CACNA1C, GABRA4, GABRA5, GABRB1, GABRB3, GABRG2, GABRG3, PTGS2, PRKCB*	7.5 × 10^−5^	1.3 × 10^−3^
Tryptophan metabolism	**7**	*ASMT, AADAT*, *DDC, MAOA, MAOB, TDO2*	2.1 × 10^−4^	3.5 × 10^−3^

Significant terms using Bonferroni correction are included. Other disease processes are not presented in this table

*KEGG* Kyoto encyclopedia of genes and genomes, *GABA* γ-aminobutyric acid

When the analysis was performed using the GO Biological Process (GOBP) database, several terms associated with ASD were over-represented, including social behavior, vocalization behavior, learning, and memory. Some of the genes associated with these terms encode neurexins (*NRXN1* and *NRXN2*), neuroligins (*NLGN4X* and *NLGN4Y*), cell adhesion molecules (*NRCAM*), and molecular scaffolding proteins that are involved in the postsynaptic density of neurotransmitter receptors (*SHANK2* and *SHANK3*. The most significant terms enriched using the GOBP database are summarized in Table 2.

**Table 2:** Results of the over-representation analysis using the GO Biological Process (BP) database

Term	#	Genes	p-value	Bonferroni
Social behavior	13	*SHANK2, SHANK3, AVPR1A, CNTNAP2, DRD3, MECP2, NRXN1, NRXN2, NLGN4X, NLGN4Y, OXTR, PTCHD1*, *SLC6A4*	1.5 × 10^−15^	2 × 10^−12^
Vocalization behavior	7	*SHANK2, SHANK3, CNTNAP2, DLG4, NRXN1, NRXN2, NLGN4X, NLGN4Y*	5.5 × 10^−10^	7.5 × 10^−7^
Adult behavior	8	*SHANK2, SHANK3, CNTNAP2, GABRG2, GRM7, NRXN1, NRXN2, NLGN4X*	8.2 × 10^−10^	1.1 × 10^−6^
Synapse assembly	10	*SHANK2, SHANK3, BDNF*, *DRD1, DRD2 MECP2, NRXN1, NRXN2, NLGN4Y, NRCA*M	9.3 × 10^–10^	1.3 × 10^−6^
Memory	10	*HTR2A, CX3CR1*, *SHANK2, SHANK3, CHRNA7, DRD1, GRIN2A, OXTR, PTGS2, SLC6A4*	1.2 × 10^−8^	1.7 × 10^−5^
Positive regulation of synaptic transmission, glutamatergic	7	*SHANK2, SHANK3, DRD1, NRXN1, OXTR, PTGS2, RELN*	4.8 × 10^−9^	6.6 × 10^−6^
Learning	9	*SHANK2, SHANK3, CNTNAP2, DRD1, DRD3, NRXN1, NLGN4X, NLGN4Y*, *PTGS2*	1.2 × 10^−8^	1.7 × 10^−5^
Vocal learning	5	*SHANK3, CNTNAP2, FOXP2*, *NRXN1, NRXN2*	1.2 × 10^−7^	1.6 × 10^−4^
Long-term synaptic potentiation	7	SHANK2, SHANK3, DRD1, GRIN2A, MECP2, RELN, SNAP25	4.3 × 10^−7^	5.9 × 10^−4^
Chemical synaptic transmission	12	*HTR2A, CACNA1G, GABRA5, GRIN2A, GRIN2B, GRIK2, GRM7, HOMER1, NRXN1, NRXN2, SLC6A4, SNAP25*	2.6 × 10^−6^	3.6 × 10^−3^

The ten most significant terms using Bonferroni correction are included

*GO* gene ontology

The cellular localization of the genes identified in this systematic review was assessed using the GO Cellular Component (GOCC) database. The over-enrichment analysis showed that the two most enriched terms were “postsynaptic membrane” (p = 1.1 × 10^−15^) and “cell junction” (p = 1.8 × 10^−11^). Other significantly enriched terms included “plasma membrane” (p = 2.6 × 10^−10^), “neuron projection” (p = 2 × 10^−8^), and “postsynaptic density” (p = 1.1 × 10^−7^).

An additional over-representation analysis was performed using the Reactome database. From the 118 initially extracted genes, 90 were found in the database, and 483 pathways were hit by at least one gene. A probability score was calculated and corrected using the Benjamini–Hochberg method for false discovery rates (FDRs) to obtain the most enriched pathways for the gene set. The ten most significant pathways, sorted by p-value, are shown in Table 3. The results obtained in this analysis were consistent with those obtained using DAVID. The enriched pathways were primarily associated with synaptic structure and function, and neurexins, neuroligins, neurotransmitter receptors, and synaptic interactions were all enriched in our gene set. Additionally, various pathways associated with transcription and synaptic regulation that are mediated by *MECP2* were also significantly enriched in this analysis.

**Table 3:** Results of the over-representation analysis using the Reactome database

Term	#	Genes	p-value
Neuronal system	28	*ABAT, ACE*, *CDH8*, *EGF*, *GABRA4, GABRA5, GABRB1, GABRB3, GABRG2, GABRG3, GRIK2, GRIN2A, GRIN2B, HOMER1, HTR3A, KCNJ10*, *MAOA, NLGN4X, NLGN4Y, NRXN1, NRXN2, PRKCB1, DLG4, SHANK2, SHANK3, SLC6A4, SNAP25, STX1A*	1.11 × 10^−16^
Neurexins and neuroligins	11	*GRIN2A, GRIN2B, HOMER1, NLGN4X, NLGN4Y, NRXN1, NRXN2, DLG4, SHANK2, SHANK3, STX1A*	6.25 × 10^−14^
Transmission across Chemical Synapses	20	*ABAT, ACE, EGF, GABRA4, GABRA5, GABRB1, GABRB3, GABRG2, GABRG3, GRIK2, GRIN2A, GRIN2B, HTR3A, KCNJ10, MAOA, PRKCB1, DLG4, SLC6A4, SNAP25, STX1A*	7.85 × 10^−12^
Protein–protein interaction at synapses	11	*GRIN2A, GRIN2B HOMER1, NLGN4X, NLGN4Y, NRXN1, NRXN2, DLG4, SHANK2, SHANK3, STX1A*	1.41 × 10^−11^
Transcriptional Regulation by MECP2	5	*BDNF, GRIN2A, GRIN2B, MECP2*, *MET*	4.91 × 10^−10^
MECP2 regulates neuronal receptors and channels	4	*GRIN2A, GRIN2B, MECP2, MET*	1.56 × 10^−9^
Neurotransmitter receptors and postsynaptic signal transmission	14	*EGF, GABRA4, GABRA5, GABRB1, GABRB3, GABRG2, GABRG3, GRIK2, GRIN2A, GRIN2B, HTR3A, KCNJ10, PRKCB1, DLG4*	1.72 × 10^−9^
GABA receptor activation	8	*EGF, GABRA4, GABRA5, GABRB1, GABRB3, GABRG2, GABRG3, KCNJ10*	2.99 × 10^−8^

The most significant pathways, according to p-value, are included

## Discussion

Multiple common genetic variants have been described among various ASD patient cohorts. Most previous studies have explored the effects of mutations in specific genes or protein alterations that may enhance our understanding of ASD. Previous studies have categorized the disorder as a synaptopathy and have attempted to describe how aberrant synapse function can contribute to neurodevelopmental disorders. [7] In this systematic review, we created a data set containing genes that have been associated with ASD in several studies worldwide. By performing an over-representation analysis of these genes, we found that genetic variants related with synaptic function, among others, may be implicated in the pathophysiology of ASD.

Genes that encode neurotransmitter receptors comprise a significant portion of our data set. In particular, several genes involved in the synthesis of GABA-A receptors were found to be altered in ASD. Associations between polymorphisms in *GABRA4*, *GABRB1*, and *GABRA2,* which are all located in the *4p12* region, were reported in Argentinian, [16] Caucasian American, [17] and African American populations. [18] Haplotype formation and interactions with other genes, such as *GABRB2* in chromosome 5, were also identified [16, 18]. Moreover, SNPs and mutations in *GABRB3*, *GABRA5*, and *GABRG3,* which are located in the *15q11–q13* region, were found to be associated with ASD in Korean and American populations [19–22]. Although many of these polymorphisms are intronic and their biological significance is not yet completely understood [16], the disruption of transmembrane chloride transport may significantly impact neuronal excitability and neurodevelopment [23]. Some previously described SNPs and mutations associated with the identified genes and chromosomal regions have also been associated with other neurological conditions, including epilepsy, Angelman syndrome (AS), and fragile-X syndrome [23].

The transmission disequilibrium test (TDT) revealed significant preferential transmission for SNPs in the *GRIK2* gene in European (rs2518261) and Korean (rs3213607) populations [24, 25]. This gene encodes a subunit of glutamate kainate receptors and has been associated with synaptic plasticity, memory, and learning [24, 25]. The disruption of glutamatergic receptors was also demonstrated in studies of *GRIN2A* and *GRIN2B*. A study examining 19 polymorphisms in *GRIN2B* reported the significant transmission and linkage disequilibrium (LD) of various SNPs and one haplotype among the Chinese Han population [26]. Moreover, an intronic polymorphism in *GRIN2A* was also preferentially transmitted during the TDT test in a European sample [27]. These genes encode subunits of N-methyl-D-aspartate (NMDA) receptors, which are responsible for sodium transport across the postsynaptic plasma membrane and have been associated with intellectual disability and epilepsy [28]. Additionally, a common genetic variation in the *GRM7* gene (rs779867), which encodes a metabotropic glutamatergic receptor, was also preferentially transmitted among Irani patients with ASD [29].

The term “serotonergic synapse” was also significantly enriched in our over-representation analysis using the KEGG pathway database. SNPs in *HTR2A* (rs6311 and rs6313) were significantly associated with ASD in Korean and Croatian populations, and an SNP in *HTR3A* (rs1150220) was identified in an American population [30–32]. The polymorphisms rs6311 and rs6313 have been extensively studied in association with several neuropsychiatric conditions, including schizophrenia and Alzheimer’s disease [33, 34]. Although their influence on ASD development remains unclear, previous studies have suggested a possible role because the 5HT-2A receptor is the primary excitatory serotonin receptor subtype, suggesting that these SNPs may also be associated with cognitive processes, such as attention, learning, and memory [35].

The identification of hyperserotonemia in patients with ASD has also led to the investigation of the long and short variants in the 5-HTT-linked polymorphic region (5HTTLPR) of the *SLC6A4* gene. This gene encodes the sodium-dependent serotonin transporter (SERT), which is a presynaptic protein that allows for the reuptake of serotonin that is released into the synaptic cleft [31, 36–39]. These studies found the increased transmission of either the short (S) or the long (L) variant, depending on the population being studied. In Korean and Israeli children, the preferential transmission of the L allele was identified, with significant LD, and the L/L genotype was associated with ASD [31, 39]. In contrast, among South African, Indian, Irish, and American children, the increased transmission of the S/S genotype and the S allele were observed [36, 38, 40, 41]. Several other genetic variants in the *SLC6A4* gene that might confer an increased risk for ASD have been reported. The rs6355 variant (Gly56Ala) was identified in American subjects [42], SNPs in the 3’ untranslated region (UTR) were found in an Indian population [41, 43], and a variable number tandem repeat (VNTR) in intron 2 was reported in Irish patients [40].

The dopaminergic synapse was one of the terms enriched in the over-representation analysis that was performed using the KEGG pathways database. Several ASD-related genes were associated with this type of synapse, including *DRD1* and *DRD3*, which encode the D1 and D3 dopamine receptors, respectively [44, 45]. In a North American population, the rs265981-C, rs4532-A, and rs686-T alleles, as well the C-A-T haplotype, for the *DRD1* gene, were over-transmitted from mothers to affected sons in the TDT analysis and were associated with social interaction and nonverbal communication difficulties [45]. Similarly, in a European population examining Dutch and British subjects, a significant association was identified between the rs167771 SNP in the *DRD3* gene and the presence of ASD [44]. Common genetic variations in genes encoding voltage-dependent calcium channels, such as *CACNA1C* and *CACNA1G,* were also found in a Chinese Han and an American population, respectively [46, 47].

An increased risk of ASD has also been associated with polymorphisms in the *OXTR* gene, which encodes the oxytocin receptor. Multiple SNPs and haplotypes on *OXTR* have been described in American [48], Chinese Han [49], Israeli [50], and Japanese [51] samples. Interestingly, Hernandez et al. identified reduced functional connectivity between the nucleus accumbens and other areas of the reward circuit as a function of increased *OXTR* risk-allele dosage in patients with ASD [48]. Additionally, Lerer et al. reported a significant association between various *OXTR* SNPs and haplotypes and ASD, IQ, and the Vineland Adaptive Behavior Scales, which suggested that genetic variations in the *OXTR* gene may play roles in several cognitive processes required for daily living skills [50]. These results are consistent with the enrichment of the *OXTR* in the terms “social behavior” and “memory” in our over-representation analysis using the GOBP database.

Several genes involved in neurotransmitter metabolism were identified in the over-representation analysis using both the KEGG pathway and Reactome databases. The *DDC* gene encodes the aromatic L-amino acid decarboxylase (*AADC*) or DOPA decarboxylase, which is important for decarboxylation reactions that occur during the formation of dopamine, histamine, and serotonin. A recent study performed in a northern Spanish sample found a significant association between ASD and both the rs6592961 SNP and a four-marker haplotype in this gene [52]. Genetic variations in the monoamine oxidase genes *MAOA* and *MAOB* have also been reported [53, 54]. In particular, a haplotype composed of three SNPs in the *MAOA* gene was significantly associated with ASD in a Korean sample [54], whereas the rs2283727 and rs2283728 SNPs in the *MAOB* gene were identified in Indian ASD patients [53]. The *MAOB* SNPs were also associated with increased platelet 5-HT levels and CARS scores for specific behavioral symptoms [53]. The significance of these genetic variants have yet to be fully elucidated. However, these genes have also been implicated in several other neuropsychiatric conditions, and these associations provide further insight into the roles played by metabolic pathways in the pathophysiology of ASD [55–57].

**Fig. 2:**
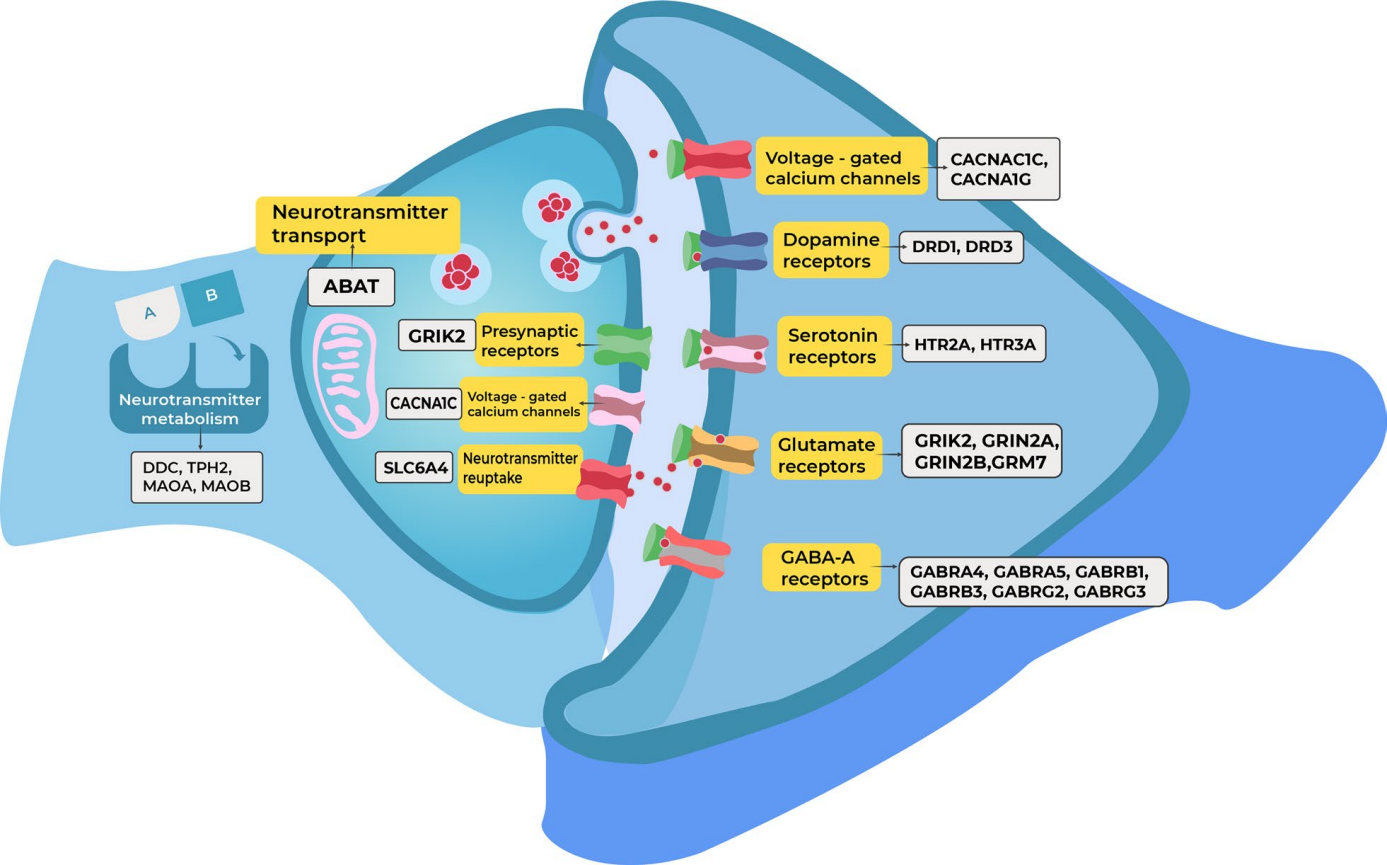
Schematic representation of the genes and biological processes affected in autism spectrum disorder. Source: Authors’ elaboration

A candidate-gene screening study performed in a European sample revealed two SNPs in the *ABAT* gene, which encodes the GABA-catabolizing enzyme 4-aminobutyrate aminotransferase, that were significantly associated with ASD in both simplex and multiplex families [27]. In addition, the C677T and A1298C variants in the *MTHFR* gene were significantly associated with ASD in three different populations of Indian, Chinese, and Canadian American descent [51, 58, 59]. The *MTHFR* gene encodes methylenetetrahydrofolate reductase, an enzyme that is involved in the metabolism of folate and the conversion of homocysteine into methionine [60]. These variants have been widely studied in association with mild hyperhomocysteinemia and increased cardiovascular risk [60–62], but they also appear to be involved in the maintenance of brain structure and function and may also be associated with ASD [63]. A graphic representation of the ASD-related genes associated with neurotransmitter receptors, metabolism, and other synaptic functions is illustrated in Fig. 2.

Tryptophan metabolism was one of the most significantly enriched terms in the over-representation analysis performed in this study. The rs375910 SNP in the tryptophan 2,3-dioxygenase gene (*TDO2*) was over-transmitted in patients with ASD from an American sample [64]. Similarly, two intronic SNPs (rs4341581 and rs11179000) in the tryptophan hydroxylase-2 gene (*TPH2*) were significantly associated with ASD in a case–control study performed among American individuals of European descent [65]. The rs5989681 and rs4446909 SNPs and several haplotypes in the *ASMT* gene were associated with ASD and autistic-like traits in two European samples [66, 67]. The *ASMT* gene encodes for the final enzyme required for melatonin biosynthesis, which suggests that these findings may partially explain the association between ASD and sleep disorders [66, 67]. Altogether, these results indicate the existence of various defects in tryptophan metabolism and may help explain the altered serotonin levels in patients with ASD, since tryptophan is a serotonin precursor. However, further investigations regarding these polymorphisms remain necessary as some of these associations have failed to be replicated in larger samples, such as those reported for the *TPH2* gene [68].

Another significant proportion of our gene set is represented by synaptic cell-adhesion molecules, including neurexins (NRXNs) and neuroligins (NLGNs). These molecules mediate signaling between pre- and postsynaptic specializations and, thus, play a significant role in information processing. Alterations in these genes have been associated with ASD and other cognitive diseases [69]. *CNTNAP2* encodes a member of the NRXN family, and its association with ASD has been widely studied, especially rs7794745, which was found to be associated with ASD in Iranian and Brazilian patients [70, 71]. This SNP was also identified as a component of haplotype T–A (rs7794745–rs10500171) and haplotype A–T–A (rs10244837–rs7794745–rs10500171), which were both associated with ASD in a Chinese Han sample [72]. In our over-expression analysis, the *CNTNAP2* gene was found in the terms “social behavior,” “learning,” “vocalization behavior,” and “vocal learning,” which are all consistent with some of the most important features of ASD.

Contactin-associated-proteins, such as that encoded by *CNTNAP2,* play major roles in extrasynaptic neuron-glia interactions, and NRXNs and NLGNs form a trans-synaptic complex that is essential for the appropriate formation and development of synapses. Interactions between presynaptic NRXNs and postsynaptic NLGNs mediate trans-synaptic cell adhesion and shape synaptic efficacy and plasticity [69]. Common genetic variations in *NRXN1*, *NRXN2*, and *NRXN3* were evaluated in two cohorts of Chinese Han patients, which revealed significant associations between ASD and the rs2303298 T allele in *NRXN1* and rs12273892 AT genotype in *NRXN2* [73, 74]. The study by Wang et al. also found that the rs12879016 polymorphism in the *NRXN3* gene might play a significant protective role in ASD [74]. In contrast, in a European population, a haplotype formed by six polymorphisms on the *NLGN4X* gene was significantly associated with ASD (Odds Ratio = 3.58) [75]. Various point mutations that affect the NRXN and NLGN genes were also reported in European and Chinese patients with ASD, suggesting that the genetic influences of these cellular adhesion molecules in ASD may be derived from both less disruptive polymorphisms and more disruptive missense mutations that affect protein structure and function [75–77].

Scaffolding proteins encoded by the SHANK gene family play a significant role in postsynaptic organization through the formation of complexes containing postsynaptic receptors, ion channels, NRXNs, and NLGNs [78, 79]. *SHANK2* and *SHANK3* have been proposed as candidate genes in ASD, and *SHANK3* mutations have been described at a frequency of 1% in ASD cases [80]. These genes are present in most of the enriched terms in our biological processes over-expression analysis (Table 2). High genetic variations in *SHANK3* were identified in the Autism Genetic Resource Exchange sample, together with *HOMER1* variants. These molecules interact and regulate metabotropic, NMDA, and AMPA glutamate receptors, mediating synaptic plasticity [81]. In addition, CNVs in region *22q13.3* involving *SHANK3* dosage were found in three Taiwanese patients: a de novo terminal deletion of approximately 106 kb at *22q13.33*, a de novo interstitial duplication of approximately 1.8 Mb at *22q13.32–q13.33*, and a microdeletion of approximately 147 kb at *22q13.33 *[82]. Another study involving Italian and US ASD patients found five potentially pathogenic alterations, resulting in a mutation rate of 2.3%, which was twice the previously reported frequency of deleterious *SHANK3* mutations [83]. Fewer studies were identified examining *SHANK2*; however, the SNP rs7671730 in this gene has been reported to be associated with ASD in a Chinese sample, and multiple haplotypes containing more than 5 SNPs have also been significantly associated with ASD risk [84].

Signaling molecules have also been studied as candidate genes for ASD, such as *RELN,* which encodes reelin, a glycoprotein related to neuronal migration that is found in the developing brain [85]. Various SNPs have been described in this gene, with variations among populations due to ethnic differences [86]. However, common variations include rs736707, which was associated with ASD risk in a Caucasian cohort [87] and among South African ASD patients [88]. Moreover, in a Chinese Han sample, the CC haplotype, composed of rs736707–rs2229864, was significantly associated with ASD [89]. Persico et al. found that longer triplet repeats in the 5’UTR conferred an increased vulnerability to ASD in Italian and US patients [90], and similar results were later described by Skaar et al. [91].

The mechanisms and molecular interactions underlying the pathophysiology of ASD remain incompletely understood. However, by analyzing the genes and biological pathways identified in this systematic review it is possible to provide insight into some of the functions and cellular mechanisms involved. Polymorphisms in genes encoding glutamatergic receptors and calcium-regulated ion channels may be associated with increased excitotoxicity in patients with ASD. This mechanism has previously been proposed as a major contributor to synaptic dysfunction through mitochondria-mediated cell damage in ASD [92]. Impaired GABAergic signaling resulting from genetic variation in GABA receptors may contribute to synaptic dysfunction through excitation-inhibition imbalance. Moreover, excitatory GABAergic signaling during the embryonic and early postnatal stages regulates neuronal migration and synapsis development, which appear to be impaired in patients with ASD [93].

Early brain overgrowth and defects in the formation of neural circuits have been observed in ASD. This association may be explained by genetic variation in genes associated with synaptic structure and development such as scaffolding molecules and cell adhesion molecules [93]. Neurexin-neuroligin interactions have been shown to promote neurotransmitter clustering and cytoskeletal polymerization that promotes adequate synaptic structures in the developing brain [94]. Evidence from animal models has shown that Nrxn1α, Shank3, and Nrxn2α knockout mice display autism-related behaviors, which may be explained by modifications that favor synaptic dysfunction [95, 96].

Glial cells also appear to play an important role in the pathophysiology of ASD. Evidence of increased microglial density in the cerebral cortex and increased levels of proinflammatory molecules in subjects with ASD support the association between microglial activation and neuronal dysfunction [97]. Although proinflammatory pathways were not significantly enriched in the over-representation analysis, several genes that are preferentially expressed in glial cells were identified. This includes genes involved with neurotransmitter metabolism such as *ABAT, MAOA,* and MAOB, as well as genes that encode for scaffolding proteins and transcriptional regulators. Furthermore, the enrichment of calcium signaling pathways and the identification of genes that encode for NMDA and metabotropic glutamate receptors suggests that disruption of synaptic modulation and plasticity via gliotransmitters may also be impaired in ASD [98].

## Conclusions

The pathophysiology of ASD involves multiple genetic, environmental, and developmental influences. Although the specific mechanisms that drive ASD remain unclear, the current understanding of the genetic variants involved in this condition point towards synaptic dysfunction as one of those mechanisms. [3, 93] In this systematic review, we used an over-representation analysis to identify and describe some of the biological processes and molecular pathways involved in the pathophysiology of ASD. Many of the genetic variants described in our review can be found in one of three groups of genes that may be involved in the pathophysiology of ASD: (1) neurotransmitter receptors, (2) genes involved in neurotransmitter metabolism, and (3) cell adhesion molecules and scaffolding proteins.

We described SNPs associated with all major synapse types, including serotonergic, dopaminergic, GABAergic, and glutamatergic synapses. Moreover, we discussed genetic variants in the gene encoding the oxytocin receptor, which appears to play a significant role in ASD [49, 50, 99]. Genes involved in the metabolism of neurotransmitters, especially tryptophan metabolism, which results in serotonin synthesis, also appear to play key roles in the development of ASD. Altered genes in the metabolic pathways of neurotransmitters may explain various findings in ASD, including hyperserotonemia [31, 36–39] and sleep disorders, due to the altered production of melatonin [66, 67]. These hypotheses have encouraged research projects with potential therapeutic alternatives, such as the use of melatonin, although no significant benefit of this intervention has been identified [66, 67]. Recently, genes that encode cell adhesion molecules and scaffolding proteins have been among the most studied. Knowledge of the function and biological significance of these molecules continues to evolve, and their importance in the adequate formation and development of synapses is increasingly recognized [69].

Among the limitations of this systematic review, some of the populations examined were studied in several articles; as a result, overlapping in the subjects of two or more studies may have occurred. Due to the heterogeneity and complexity of genetic data, further statistical analysis was not performed. Our search was directed towards case-series and population-based studies aiming to identify genetic variation in different populations and use primary data from clinical studies. The scope of this review was to describe and explore some of the pathways involved in autism that may be associated with common genetic variation. Therefore, the findings described in this review suggest that encouraging further investigation into synaptic dysfunction may be critical for achieving a better understanding of ASD.

## Supplementary Information

**Additional file 1.** Database of genes associated with ASD in this systematic review.

## Data Availability

The datasets generated and analyzed in this study can be found in the supplemental data to enable others to perform similar studies and replicate our findings. Further information is available from the corresponding author under reasonable request.
